# Differential Modulating Effect of Acupuncture in Patients With Migraine Without Aura: A Resting Functional Magnetic Resonance Study

**DOI:** 10.3389/fneur.2021.680896

**Published:** 2021-05-28

**Authors:** Shanshan Liu, Shilei Luo, Tianwei Yan, Wen Ma, Xiangyu Wei, Yilei Chen, Songhua Zhan, Bo Wang

**Affiliations:** ^1^Department of Acupuncture and Moxibustion, Shuguang Hospital Affiliated to Shanghai University of Traditional Chinese Medicine, Shanghai, China; ^2^Department of Radiology, Shuguang Hospital Affiliated to Shanghai University of Traditional Chinese Medicine, Shanghai, China

**Keywords:** acupuncture, migraine without aura, resting-state functional magnetic resonance imaging, regional homogeneity, cerebellum, angular gyrus

## Abstract

**Introduction:** Migraine is a recurrent neurological disorder, the symptoms of which can be significantly relieved by acupuncture. However, the central mechanism *via* which acupuncture exerts its therapeutic effect in migraine is unclear. The aim of this study was to compare the differences in regional homogeneity (ReHo) between patients with migraine without aura (MwoA) and healthy controls (HCs) and to explore the immediate and cumulative therapeutic effect of acupuncture in patients with MwoA using resting-state functional magnetic resonance imaging (fMRI).

**Methods:** The study subjects were 40 patients with MwoA and 16 matched HCs. The patients with MwoA received acupuncture on 2 days per week for 6 weeks for a total of 12 sessions followed by 24 weeks of follow-up. The primary clinical efficacy outcomes were the number of days with migraine and the average severity of headache. Secondary outcomes were the Migraine-Specific Quality of Life Questionnaire, Self-Rating Anxiety Scale, and Self-Rating Depression Scale scores. In the migraine group, resting-state blood-oxygen-level-dependent fMRI scans were obtained at baseline and after the first and 12th acupuncture sessions to measure the ReHo value. In the HCs, only a baseline resting-state blood-oxygen-level-dependent fMRI scan was obtained.

**Results:** Compared with the control group, the migraine group had a significantly lower ReHo value in the cerebellum, which increased after the first acupuncture session. Long-term acupuncture significantly improved migraine symptoms and mood with a therapeutic effect that lasted for at least 6 months. After 12 acupuncture sessions, there were significant increase of cerebellum and angular gyrus in the migraine group.

**Conclusion:** These findings suggest that migraine is related to cerebellar dysfunction. Acupuncture can relieve the symptoms of migraine, improve dysfunction of cerebellum, and activate brain regions involved in modulation of pain and emotion The cumulative therapeutic effect of acupuncture is more extensive and significant than its immediate effect.

## Introduction

Migraine is a common neurological disorder that manifests as recurrent, unilateral, moderate or severe, throbbing, pulsating headaches which lasting from several hours to several days. The headache is often accompanied by nausea or vomiting and sensitivity to sound or light and is triggered by tiredness, changes in weather, and fluctuations in mood (1, 2). Migraine is an important social and public health issue because of its high prevalence, its substantial economic burden, and the limitations it imposes on daily life (3). The medications used to prevent and treat migraine have limited efficacy and multiple adverse effects, including weight gain, fatigue, sleep disturbance, gastrointestinal upset, and medication overuse–induced headache (4, 5). Acupuncture is widely used as a non-pharmacological treatment for migraine in China and Western countries. Many large multicenter randomized clinical trials have confirmed the beneficial effects of acupuncture for migraine. Acupuncture can significantly relieve pain during an acute episode of migraine, attenuate future episodes by reducing the frequency, severity, and duration of headaches, and improve quality of life (6–9).

In recent years, with the advent of neuroimaging technology, functional magnetic resonance imaging (fMRI) has become a powerful tool that can map intrinsic brain activity and could potentially elucidate the neural mechanisms of migraine. Several fMRI studies have confirmed that migraine is a central nervous system disorder. Structural and functional alterations have been documented in the insula, anterior cingulate cortex, thalamus, prefrontal cortex, parahippocampal cortex, periaqueductal gray matter, and cerebellum, and frequent episodes of migraine may lead to further damage in pain-related resting-state brain networks over time (10–12). There is evidence showing that acupuncture can modulate pain-related brain regions and neural activity with distinct patterns at different periods (13, 14). Therefore, we believe that fMRI could be used as a tool to investigate the neural responses to acupuncture in patients with migraine. Thus far, the fMRI studies that have investigated the therapeutic effect of acupuncture in migraine have mostly been limited to before and after comparisons, with no research on the effect of acupuncture in different periods of treatment.

Given that patients with migraine have multiple functional and structural abnormalities and that acupuncture is an effective treatment for migraine, we performed a study in which we compared the regional homogeneity (ReHo) (15) in patients with migraine without aura (MwoA) with that in healthy controls (HCs). All patients with MwoA in that study received 6 weeks of standard acupuncture treatment and were followed up for 24 weeks thereafter to assess the effects of treatment. In order to explore the modulating effect of acupuncture, we obtained fMRI scans on three occasions, namely, at baseline and after the first and 12th acupuncture sessions. The relationship between change in ReHo and improvement in migraine symptoms was also examined. We then hypothesized that there could be a difference in ReHo values between patients with MwoA and HCs in that acupuncture may have different modulating effects by improving dysfunctional brain regions and activating brain regions related to pain and emotional modulation. The purpose of the present study was to compare the differences in ReHo values between patients with MwoA and HCs and to explore the immediate and cumulative therapeutic effect of acupuncture in patients with MwoA using resting-state fMRI.

## Materials and Methods

### Participants

Forty patients with MwoA were recruited from the neurology outpatient clinic or acupuncture clinic at the Shuguang Hospital affiliated to Shanghai University of Traditional Chinese Medicine (16). MwoA was diagnosed based on the International Classification of Headache Disorders, 3rd Edition ICHD-III criteria (17). The inclusion criteria were as follows: age 18–65 years, right-handedness, 2–8 migraine attacks during the past month, diagnosis of migraine at least 6 months earlier, no prophylactic headache medications during the past month, no psychoactive or vasoactive agents during the past 3 months, no history of acupuncture, and ability to provide informed consent. Sixteen right-handed HCs matched for age and education level were recruited by advertisements and required to have a normal neurological examination. The following exclusion criteria were applied: other type of primary or secondary headache, history of a clinically significant disorder, pregnancy or breast-feeding, contraindications to MRI or acupuncture, severe head deformity, and intracranial lesions.

The study protocol was approved by the Ethics Committee of the Shuguang Hospital affiliated to Shanghai University of Traditional Chinese Medicine and is registered on www.chictr.org.cn (ChiCTR1900023105). Informed consent was obtained from all study participants.

### Study Design

The patients with MwoA were observed for a total of 34 weeks. Weeks −4 to 0 served as a baseline phase, during which the headache status at baseline was recorded. Weeks 1–6 served as an intervention phase, during which patients with migraine received standard acupuncture treatment. Weeks 7–30 served as the follow-up period when headache status was monitored. All the patients kept a headache diary throughout the study period. fMRI scans were obtained from the patients with MwoA before and after the first and 12th acupuncture sessions immediately (all fMRI scanned within 1 h before and after acupuncture). All patients with MwoA had been migraine-free for at least 72 h at the time of the fMRI scans. Only a baseline fMRI scan was obtained from the HCs.

### Interventions

All patients received 12 sessions of acupuncture, consisting of 2 sessions per week, each lasting 20 min for 6 weeks. Acupoints were selected according to the Chinese guidelines for acupuncture in patients with migraine and those used in previous randomized controlled trials. The following acupoints were selected: Baihui (DU20), Taiyang (EX-HN5), bilateral Fengchi (GB20), Shuaigu (GB8), Xuanlu (GB5), Toulinqi (GB15), Hegu (LI4), and Taichong (LR3) (6, 18). Two licensed acupuncturists administered all the acupuncture treatments. Sterile, single-use acupuncture needles with a length of 25–40 mm and a diameter of 0.25 mm were inserted to achieve the Deqi sensation (19). Electric stimulation was applied at GB20 and GB8 bilaterally; the stimulation frequency was 2 Hz and the intensity was varied from 0.1 to 1.0 mA until the patient felt comfortable (20). All patients agreed not to take any regular medications for migraine for the duration of the study. In the event of severe pain, ibuprofen (as 300-mg sustained-release capsules) was allowed as rescue medication.

### Outcome Measures

The primary efficacy outcomes were the number of days with migraine attacks and average severity of headache [visual analog scale (VAS) pain score] and the secondary outcomes were and the Migraine-Specific Quality of Life Questionnaire (MSQ), Self-Rating Anxiety Scale (SAS), and Self-Rating Depression Scale (SDS) scores. Headache status in the previous 4 weeks was evaluated before treatment (week 0), at the end of treatment (week 6), and at follow-up 1 month (week 10), 3 months (week 18), and 6 months (week 30) later. Researchers recorded all acupuncture treatments and the reasons for dropping out during the study. Acupuncture-related adverse events, including bleeding, subcutaneous hemorrhage, severe pain, fainting, and local infection, were recorded at each treatment session.

### Acquisition of fMRI Data

All fMRI scans were obtained at the Shuguang Hospital MRI Center using a 3.0-T MRI scanner (uMR780 Platform, United Imaging Medical Systems, Shanghai, China) with a 12-channel flexible head coil. All subjects were required to stay awake, remain motionless, and keep their eyes closed and ears plugged during the scan. A high-resolution structural image for each subject was acquired using a three-dimensional MRI sequence with a voxel size of 1 mm^3^ employing an axial fast-spoiled gradient-recalled sequence (repetition time, 7.2 ms; echo time, 3.1 ms; slice thickness, 1.0 mm; flip angle, 10°; field of view, 256 × 256 mm; and data matrix, 256 × 256). Eight-minute blood-oxygen-level-dependent resting-state fMRI scans were obtained with echo-planar imaging (30 contiguous slices with a slice thickness of 3.5 mm, a repetition time of 2,000 ms, an echo time of 30 ms, a slice thickness of 3.5 mm, a flip angle of 90°, a field of view of 240 × 240 mm, and a data matrix of 64 × 64).

### Clinical Data Analysis

The statistical analysis was analyzed IBM SPSS Statistics for Windows 22.0 (IBM Corp., Armonk, NY). The baseline characteristics and clinical outcomes were presented as mean (standard deviation) and categorical variables as the number (percentage). Chi-square was applied for categorical variables comparisons. Two sample *t*-test/Mann-Whitney *U* were applied for two-group comparisons, and one-way ANOVA was applied when there were multiple assessment points. All tests were two-tailed and a *P*-value of <0.05 was considered statistically significant.

### MRI Data Collection

The fMRI data were preprocessed and analyzed using SPM12 (https://www.fil.ion.ucl.ac.uk/spm/software/spm12/) in MATLAB. The main steps were as follows: (1) the first 10 volumes of each scan were removed to avoid instability due to a T1-related relaxation effect; (2) slice timing, to correct the time difference between the data at each point in time; (3) realigning, the data at all time points were spatially aligned with the data collected at the first time point to obtain the head motion parameters of the subject in the scanning time series [with excessive motion (>3 mm) were discarded]; (4) normalization, all the data collected were resampled according to the Montreal Neurological Institute (MNI) standard template space with a 3 × 3 × 3 mm voxel size for spatial normalization; (5) detrending, mean signals from white matter and cerebrospinal fluid were regressed out, leaving the gray matter signal for denoising; (6) filtering, the band-pass filtering range was set at 0.01–0.08 Hz to physiological and high frequency noise; and (7) smoothing, conducted on final ReHo maps with a Gaussian kernel of 6 mm full width at half-maximum.

### ReHo Value Analysis

Group analysis was performed with a random effects model using SPM12. We first compared the difference in ReHo between patients with MwoA and HCs using two-sample *t*-test. We then compared the changes in ReHo in different stages during acupuncture treatment using one-way ANOVA within subjects; *post-hoc* comparisons were used to test the changes in ReHo between different periods. To explore the association between clinical outcomes and the ReHo value, we extracted the *Z*-values for each patient in the regions of interest, in which patients with MwoA showed a decreased ReHo value before acupuncture when compared with HCs and a significantly increased ReHo value after acupuncture, and used Pearson/Spearman correlation to explore the association between these brain regions and the corresponding migraine symptoms. A threshold of family-wise error (FWE) corrected *P*-value of <0.05 at the voxel level or at the cluster level were applied for all analyses.

## Results

**Figure 1:**
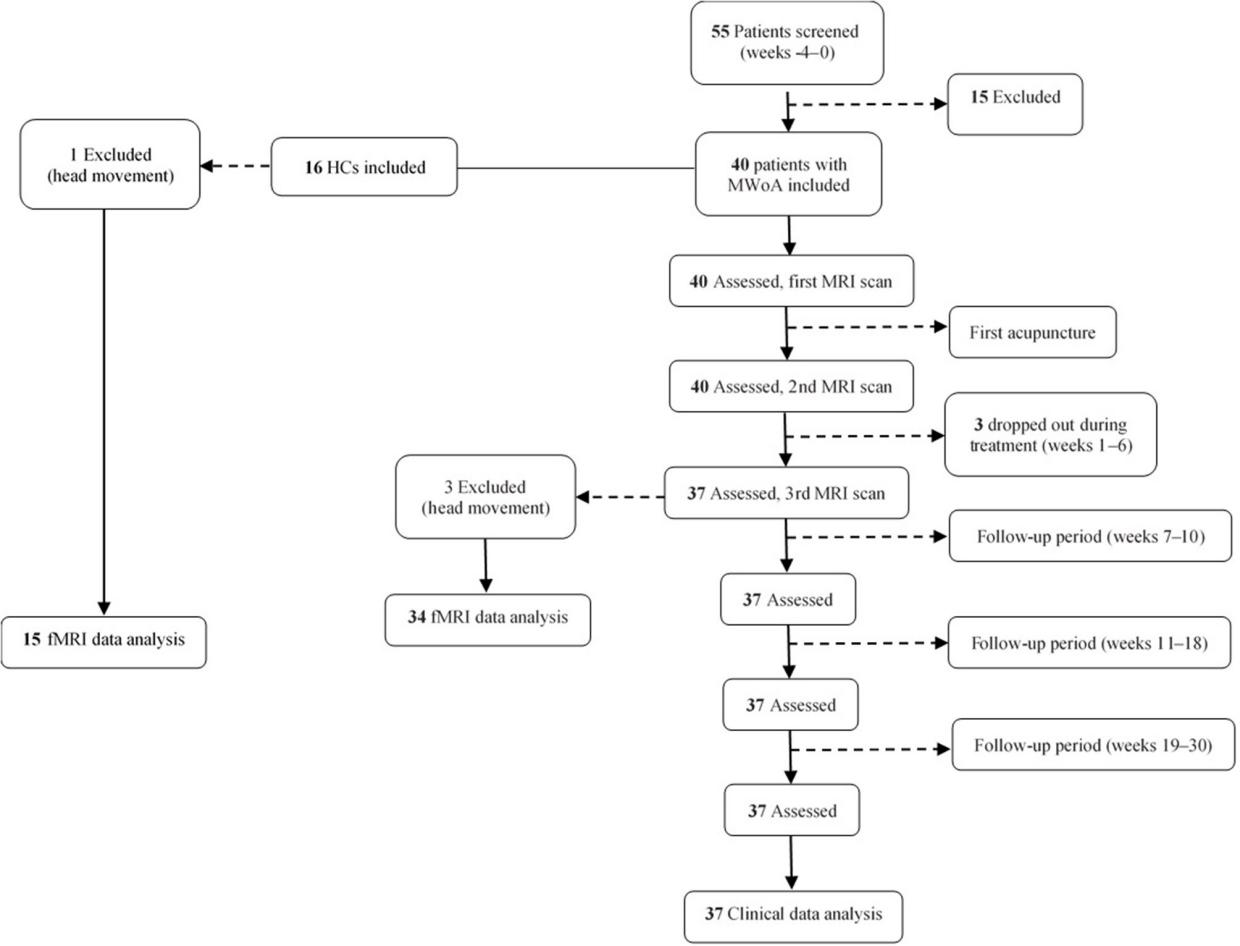
Flow chart showing the screening, enrollment, treatment, and follow-up periods. fMRI, functional magnetic resonance imaging; MRI, magnetic resonance imaging; MwoA, migraine without aura.

Forty patients with MwoA and 16 HCs matched for age and sex were recruited for this study. Three patients were lost to follow-up, leaving clinical data for 37 patients available for analysis. Three patients with MwoA and one HC were excluded because of excessive head movement during scanning. Finally, fMRI data for 37 patients and 15 HCs were included in the analysis (Figure 1).

### Baseline Characteristics

There were no significant differences in demographic characteristics, including age, sex, and educational level, between the two study groups (*P* > 0.05; Table 1).

**Table 1:** Clinical characteristics of patients with migraine without aura and healthy controls.

**Characteristic**	**Patients with MWoA (*n* = 37)**	**Healthy controls (*n* = 15)**	***P*-value**
Sex (male/female)	6/31	2/13	1.00
Age (years)	37.97 (9.82)	34.88 (6.66)	0.50
Education (years)	15.03 (3.14)	15.94 (1.95)	0.30
Duration of migraine (years)	16.19 (12.81)	/	/

*Data are shown as the mean (standard deviation). Results from independent-samples t-test of the comparison between patients and healthy controls. MwoA, migraine without aura*.

### Clinical Outcomes

At the end of the acupuncture treatment, days with migraine and the average VAS score for headache severity were significantly lower than at baseline (*P* < 0.01). The restrictive, preventive, and emotional functional subscales of the MSQ in the migraine group showed significant improvement when compared with baseline (*P* < 0.01). The SAS and SDS scores were significantly improved compared with baseline in the migraine group (*P* < 0.05; Table 2).

**Table 2:** Clinical outcomes in patients with migraine without aura during the study period.

**Assessment points (wks)**	**Days with migraine**	**VAS score**	**MSQ score, restrictive subscale**	**MSQ score, preventive subscale**	**MSQ score, emotional functional subscale**	**SAS score**	**SDS score**	***P*****-value for pairwise comparison**	
								**VS baseline**	**VS treatment**
Baseline, −4–0	5.16 (1.66)	7.73 (1.41)	55.14 (14.81)	61.49 (20.91)	62.52 (23.06)	43.76 (9.33)	47.38 (9.80)		<0.001
Treatment, 1–6	1.57 (1.21)	4.08 (1.26)	75.21 (13.15)	83.38 (14.96)	83.42 (15.53)	39.73 (8.84)	40.38 (10.61)	<0.001	
Follow-up, 7–10	1.16 (0.99)	4.49 (1.48)	78.61 (13.38)	85.68 (13.75)	84.32 (14.42)	39.46 (8.24)	41.03 (11.40)	<0.001	>0.05
Follow-up, 11–18	1.54 (0.77)	4.43 (1.46)	78.07 (12.90)	85.27 (11.30)	84.50 (13.25)	39.49 (8.51)	39.89 (11.03)	<0.001	>0.05
Follow-up, 19–30	1.44 (0.70)	4.89 (1.08)	74.29 (14.00)	80.00 (12.83)	83.70 (11.70)	40.97 (7.98)	42.70 (9.62)	<0.001	>0.05

*Data are shown as the mean (standard deviation). The P-values indicate the results of one-way analysis of variance for comparison between different periods of treatment in the migraine group. MSQ, Migraine-Specific Quality of Life Questionnaire; SAS, Self-Rating Anxiety Scale; SDS, Self-Rating Depression Scale; VAS, visual analog scale*.

The effects of acupuncture seemed to be maintained during the follow-up period. Days with migraine, the VAS score, the restrictive, preventive, and emotional functional subscale scores on the MSQ, and the SAS and SDS scores in the migraine group were significantly lower at the end of acupuncture treatment than at baseline at each interview during weeks 7–30. There was no difference in any of the clinical data between weeks 10, 18, and 30.

### ReHo Results

#### Migraine Group vs. Control Group

To explore the neural pathophysiology of migraine, we first compared the ReHo values between the migraine group and the control group using two sample *t*-test. The ReHo for the cerebellum was significantly lower in the migraine group than in the HC group (*P* < 0.05, FWE-corrected at the cluster level; Table 3).

**Table 3:** Changes in regional homogeneity in patients with migraine without aura and healthy controls during the study period.

**(A) Difference in ReHo between MwoA and HCs**						
**Contrast**	**Brain region**	**MNI (x, y, z)**			***T*-value**	**Voxels**
PM < HC	Right cerebellum	18	−60	−42	4.4607	131
	Left cerebellum	−18	−63	−42	4.9429	102
**(B) Changes in ReHo in different stages of treatment**						
**Contrast**	**Brain region**	**MNI (x, y, z)**			***F*-value**	**Voxels**
PMa-PMb-PMc	Left cerebellum	−9	−36	−30	3.805	20
	Left angular gyrus	−60	−63	27	3.7857	23
**(B1) Changes in ReHo in different stages (*****post-hoc*** **analysis)**						
**Contrast**	**Brain region**	**MNI (x, y, z)**			***T*-value**	**Voxels**
PMb > PMa	Left cerebellum	−12	−33	−33	4.7578	20
**(B2) Changes in ReHo in different stages (*****post-hoc*** **analysis)**						
**Contrast**	**Brain region**	**MNI (x, y, z)**			***T*-value**	**Voxels**
PMc > PMa	Left angular gyrus	−57	−57	24	4.848	23
**(B3) Changes in ReHo in different stages (*****post-hoc*** **analysis)**						
**Contrast**	**Brain region**	**MNI (x, y, z)**			***T*-value**	**Voxels**
PMc > PMb	Left angular gyrus	−60	−63	27	3.9827	20

*(A) Comparison of ReHo between patients with MwoA and HCs. (B) Changes in ReHo in different periods of acupuncture treatment (P < 0.001, GRF, cluster P < 0.05, cluster >20). (B1) Changes in ReHo between before and after first acupuncture session (FWE, P < 0.05). (B2) Changes in ReHo between before and after acupuncture (FWE, P < 0.05). (B3) Changes in ReHo between after first acupuncture session and after 12th acupuncture session (FWE, P < 0.05). FWE, family-wise error; HCs, healthy controls; MwoA, migraine without aura; ReHo, regional homogeneity; PM, patients with migraine; PMa, before acupuncture; PMb, after first acupuncture session; PMc, after 12th acupuncture session*.

#### Modulating Effects of Acupuncture

The modulating effect of acupuncture during the different periods of treatment was explored using ANOVA within subjects. The ReHo values in the cerebellum and angular gyrus increased in the migraine group during the different periods of acupuncture treatment (*P* < 0.05, GRF-corrected at the cluster level). *Post-hoc* comparisons showed that the ReHo value increased significantly in the cerebellum after the first acupuncture session compared with baseline (FWE, *P* < 0.05) and in the angular gyrus at the end of the course of acupuncture compared with baseline and after the first acupuncture session (FWE, *P* < 0.05; Table 3).

#### Correlation Analyses Between ReHo and Clinical Outcomes

**Figure 2:**
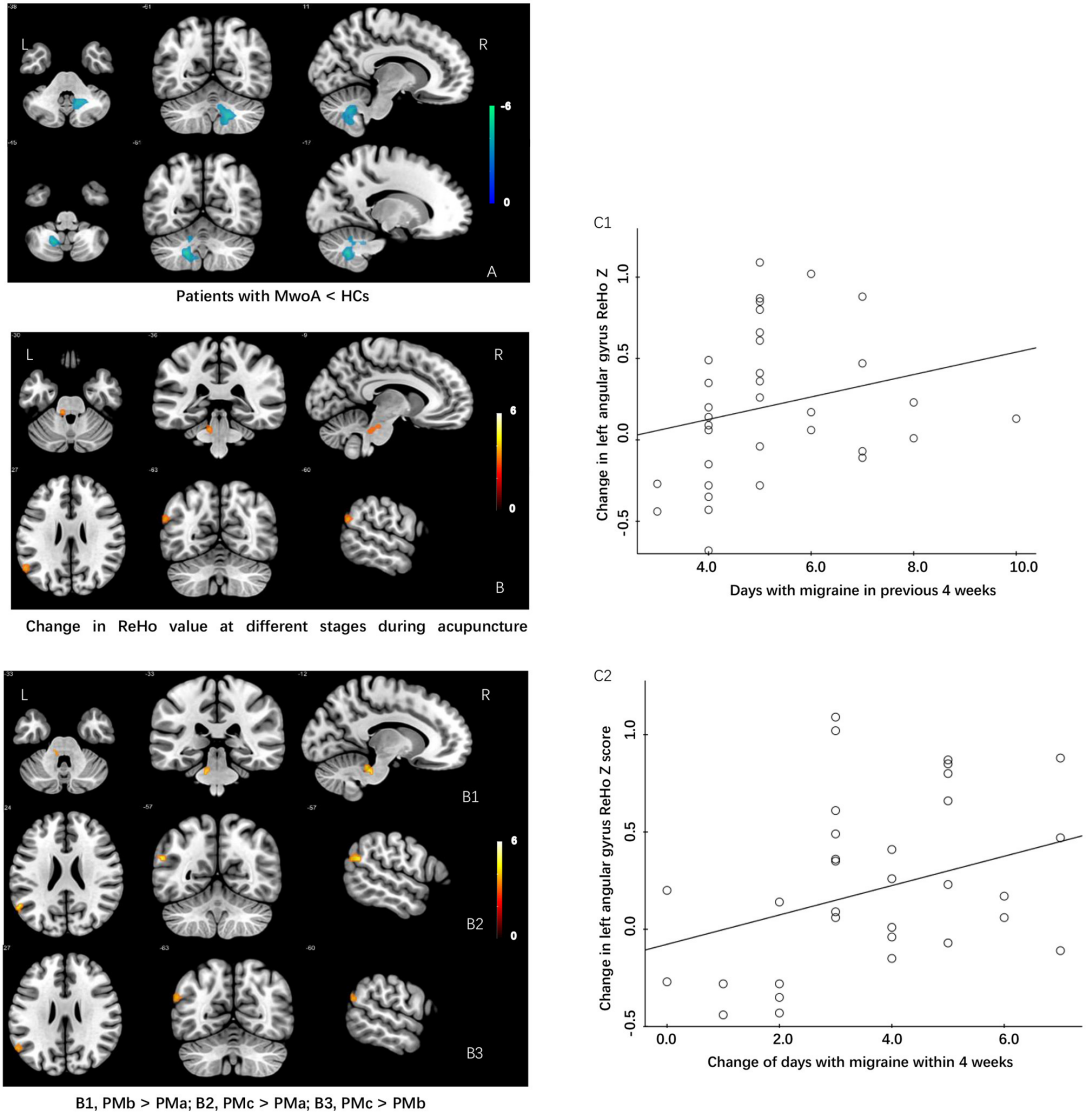
Differences in ReHo values and modulating effects of acupuncture at different periods during treatment between patients with MWoA and HCs. **(A)** ReHo value is lower in patients with MwoA than in HCs. **(B)** Increase in ReHo value during different periods of acupuncture treatment in patients with MwoA. **(B1)** Increase in ReHo value in the left cerebellum after the first acupuncture treatment compared with baseline. **(B2)** Increase in ReHo value in the left angular gyrus after the 12th acupuncture treatment compared with baseline. **(B3)** Increase in ReHo value in the left angular gyrus after the 12th acupuncture treatment compared with the first acupuncture treatment. **(C1)** The change in the ReHo value in the left angular gyrus was positively correlated with the number of days with migraine in the past 4 weeks at baseline. **(C2)** Change in the ReHo value in the left angular gyrus was positively correlated with a change in the number of days with migraine within the past 4 weeks. HCs, healthy controls; ReHo, regional homogeneity; MWoA, migraine without aura.

We extracted the mean ReHo values in the cerebellum and angular gyrus for which there were significant differences at baseline and during different periods of acupuncture treatment and performed correlation analysis with clinical outcomes (days with migraine, VAS, MSQ, SAS, and SDS scores) across all subjects. We found positive correlations of the change in ReHo value in the angular gyrus with days of migraine at baseline (*r* = 0.375, *P* = 0.029) and with the change in number of days with migraine (*r* = 0.342, *P* = 0.048). There was no significant correlation between the ReHo value for the cerebellum and any clinical outcome (Table 3 and Figure 2).

## Discussion

A migraine attack is usually unilateral and related to the circulation of the gallbladder meridian of foot Shaoyang. Based on the characteristics of migraine and past clinical experience with selection of acupoints (6, 18), we chose DU20, EX-HN5, bilateral GB20, GB8, GB5, GB15, LI4, and LR3 combined with electroacupuncture stimulation to achieve the best possible clinical effect. Previous studies have shown that acupuncture has a marked analgesic effect both immediately after an acupuncture session and during a longer-term course of acupuncture. It can reduce the frequency of migraine, days with migraine, and severity of headache to a greater extent than sham acupuncture or medication and improves quality of life and mood (21–23). This study demonstrates that acupuncture is an effective way of treating patients with MwoA in that it can significantly reduce the number of days with migraine, attenuate the severity of headache, improve quality of life, and relieve mood disorders, such as depression and anxiety. Moreover, during follow-up, the cumulative clinical effect of acupuncture was maintained for at least 6 months after the end of treatment. Overall, the therapeutic effects of acupuncture were significant, favorable, and durable.

In order to evaluate the clinical efficacy of acupuncture for migraine, we investigated the difference in ReHo between patients with MwoA and HCs and observed the immediate and cumulative effects at different periods during acupuncture treatment in the group with MwoA. To the best of our knowledge, this study is one of very few trials to have used fMRI to investigate different clinical efficacy of acupuncture. We found a difference in ReHo between patients with MwoA and HCs, namely, a decreased ReHo value in the cerebellum in patients with MwoA before the first acupuncture session and a significantly increased ReHo value after the first and 12th acupuncture session. The cerebellum is involved not only in motor coordination and learning (24, 25) but also in psychological processes, such as cognitive behavior and emotional processing (26–28), and can respond to nociceptive stimuli and participate in the management of pain (29, 30). It also plays an important role in development of migraine (31). Previous studies used voxel-based morphometry, amplitude of low frequency fluctuation, and functional connectivity to compare resting-state functional and structural abnormalities in patients with MwoA and those in HCs. It was reported that changes in amplitude of low frequency fluctuation and gray matter volume in multiple cerebellar regions in patients with MwoA were related to severity of headache and periodicity of attacks and to decreased functional connectivity with higher cognitive brain regions, such as the prefrontal lobe and posterior parietal lobe (32, 33). Amin et al. (34) observed the brain activity of drug-induced migraine by resting-state functional connectivity and found a significant decrease in the connectivity between the right cerebellum and the default network, which established that the nociceptive stimulation caused by frequent episodes of migraine affect the structure and function of the cerebellum. The dysfunction of cerebellum in patients with MwoA was caused by repeated episodes of migraine. Our results of this research showed decreased ReHo in the cerebellum which are consistent with previous reports. Russo et al. (35) suggested the diminished functional cerebellar-thalamic connectivity in patients with MwoA leading to susceptibility for migraine attacks. And cerebellum performs a control function on the descending pain modulatory system (32). Our finding proved that therapeutic effects of acupuncture treatment can modulate the dysfunction of cerebellum to improve symptoms of migraine.

Furthermore, we observed that there was a significantly greater ReHo change in the angular gyrus after 12th acupuncture sessions. The angular gyrus is a part of the inferior parietal lobule and is located in the posterior part of this lobule and the junction of the occipital, temporal, and parietal lobes. It is mainly involved in the response to pain and the sensation of temperature and pressure (36, 37). It is also an important region in the default mode network (DMN), which participates in the brain's cognitive, emotional, behavioral and other advanced functions in the resting state (38). Previous fMRI studies have found a significantly increased volume of gray matter in the angular gyrus in patients with migraine, which may be related to self-adaptation of the central nervous system, leading to abnormal brain sensitization (39). Patients with MwoA have less functional connectivity between the angular gyrus and pain and emotion related brain regions such as the cerebellum, S1, and the temporal lobe. The structural and functional changes in the key region of the DMN would impair cognition and pain management (40, 41). The angular gyrus is an important part of the DMN, and an abnormality in its structure and function affects transmission and processing of pain and emotional cognition. The finding of decreased connectivity between the angular gyrus temporal lobe and frontal lobe has been found in the initial phase of depression which proved that the angular gyrus also involved in emotional response (42, 43). In this study, the activation of the angular gyrus indicates that long-term acupuncture treatment can not only regulate abnormal cerebellar regions but also activate the angular gyrus which related pain and emotional modulation to relief painful symptoms of migraine as well as emotional disorder. Thus, with accumulation of more sessions, the cumulative modulating therapeutic effect of acupuncture can better promote the rehabilitation of migraine from more aspects, which seen on fMRI becomes more extensive and significant than the immediate effect. Pervious study observed that frequent episodic migraine showed decreased ReHo value in the angular gyrus than infrequent episodic migraine group (44). Although, the angular gyrus only showed significant increase after the 12th sessions of acupuncture and no such decrease at baseline, this may be caused by small sample sizes and days of migraine attack were relatively low which need further studies with larger sample size and larger ranges of migraine attack frequency to confirm these results.

Moreover, we found that the change of mean ReHo value in the angular gyrus which showed significant increased during different periods of acupuncture treatment positively correlated with days with migraine at baseline and the change in number of days with migraine. This suggested that patients with MwoA showed more days of migraine attacks at baseline, the greater increase in the ReHo value of the angular gyrus after treatment. And the greater reduction in number of days with migraine, the more significant positive activation of the angular gyrus. Therefore, the increased ReHo value of the angular gyrus in patients with MwoA revealed that therapeutic effects of acupuncture can modulate pain and emotional related brain regions to improve the symptoms of migraine. ReHo value Changes in the angular gyrus can be used as an objective index of clinical efficacy to confirm the effect of acupuncture in patients with migraine.

Acupuncture is a widely used form of physical and mental therapy that improves various types of pain by enhancing the interaction between endogenous analgesia, emotional processing, memory, and the DMN to rebuild a dynamic balance of psychophysical pain (45–47). Therefore, the therapeutic effect of acupuncture may be related to its ability to regulate the structure and function of these brain regions. In view of the results of this study, we believe that reversal of the ReHo value in the cerebellum and angular gyrus may have a beneficial effect in the management of painful symptoms in patients with migraine.

This study has several limitations. First, it did not include a control group that received sham acupuncture or medication, which meant that the superiority of acupuncture and the specificity of the acupoints used could not be confirmed. Second, all MRI scans were performed when the patients were not experiencing migraine, so the immediate clinical efficacy and brain activity after the first acupuncture treatment could not be assessed during an actual episode of migraine. Finally, the patients with MwoA were not followed up by fMRI scans. Therefore, any correlation between persistent changes in ReHo in the brain and clinical efficacy could not be investigated in depth. Further studies that include control groups and more frequent acquisition of MRI scans are needed.

## Conclusion

Our results demonstrate that acupuncture is an effective treatment for migraine that can significantly improve the number of days with migraine, severity of headache, quality of life, and mood and that these effects can persist for at least 6 months. Patients with migraine have abnormalities in pain-related brain regions. The immediate and cumulative effects of acupuncture are different in that acupuncture can activate abnormal brain regions immediately whereas long-term acupuncture treatment exerts an effect by activating more extensive areas of the brain. These objective findings confirm that acupuncture can reverse abnormal brain regions and activate pain and emotional modulation system to improve symptoms of migraine.

## Data Availability Statement

The original contributions presented in the study are included in the article/supplementary material, further inquiries can be directed to the corresponding author.

## Ethics Statement

The studies involving human participants were reviewed and approved by the Ethics Committee of the Shuguang Hospital affiliated to Shanghai University of Traditional Chinese Medicine. The patients/participants provided their written informed consent to participate in this study. Written informed consent was obtained from the individual(s) for the publication of any potentially identifiable images or data included in this article.

## Author Contributions

SLi drafted the manuscript and recruited the subjects. SLu and TY recruited the subjects. WM and SZ revised the manuscript. XW and YC analyzed the data. BW conceived and designed the study and revised the manuscript. All authors contributed to the article and approved the submitted version.

## Conflict of Interest

The authors declare that the research was conducted in the absence of any commercial or financial relationships that could be construed as a potential conflict of interest.

## Abbreviations

fMRI: functional magnetic resonance imaging
HCs: healthy controls
MSQ: Migraine-Specific Quality of Life Questionnaire
MwoA: migraine without aura
ReHo: regional homogeneity
SAS: Self-Rating Anxiety Scale
SDS: Self-Rating Depression Scale.

## References

[1] Santangelo G Russo A Trojano L Falco F Marcuccio L Siciliano M. Cognitive dysfunctions and psychological symptoms in migraine without aura: a cross-sectional study. J Headache Pain. (2010) 17:76. 10.1186/s10194-016-0667-0 27568039 PMC5002274

[2] Schwedt TJ. Multisensory integration in migraine. Curr Opin Neurol. (2013) 26:248–53. 10.1097/WCO.0b013e328360edb1 23591684 PMC4038337

[3] Lipton RB Bigal ME Diamond M Freitag F Reed ML Stewart WF. Migraine prevalence, disease burden, and the need for preventive therapy. Neurology. (2007) 68:343–9. 10.1212/01.wnl.0000252808.97649.21 17261680

[4] Diener H-C Charles A Goadsby PJ Holle D. New therapeutic approaches for the prevention and treatment of migraine. Lancet Neurol. (2015) 14:1010–22. 10.1016/S1474-4422(15)00198-2 26376968

[5] Evans RW Linde M. Expert opinion: adherence to prophylactic migraine medication. Headache. (2009) 49:1054–8. 10.1111/j.1526-4610.2009.01471.x 19583596

[6] Zhao L Chen J Li Y Sun X Chang X Zheng H. The long-term effect of acupuncture for migraine prophylaxis: a randomized clinical trial. JAMA Intern Med. (2017) 177:508–15. 10.1001/jamainternmed.2016.9378 28241154

[7] Xu S Yu L Luo X Wang M Chen G Zhang Q. Manual acupuncture versus sham acupuncture and usual care for prophylaxis of episodic migraine without aura: multicenter, randomized clinical trial. BMJ. (2020) 368:m697. 10.1136/bmj.m697 32213509 PMC7249245

[8] Wang LP Zhang X-Z Guo J Liu H-L Zhang Y Liu C-Z. Efficacy of acupuncture for acute migraine attack: a multicenter single blinded, randomized controlled trial. Pain Med. (2012) 13:623–30. 10.1111/j.1526-4637.2012.01376.x 22536889

[9] Li Y Liang F Yang X Tian X Yan J Sun G. Acupuncture for treating acute attacks of migraine: a randomized controlled trial. Headache. (2009) 49:805–16. 10.1111/j.1526-4610.2009.01424.x 19438740

[10] Kruit MC Vanbuchem MA Hofman PA Bakkers JT Terwindt GM Ferrari MD. Migraine as a risk factor for subclinical brain lesions. JAMA. (2004) 291:427–34. 10.1001/jama.291.4.427 14747499

[11] Schmitz N Admiraal-Behloul F Arkink E B Kruit MC Schoonman GG Ferrari MD. Attack frequency and disease duration as indicators for brain damage in migraine. J Head Face Pain. (2008) 48:1044–55. 10.1111/j.1526-4610.2008.01133.x 18479421

[12] Schwedt TJ Chong CD. Functional imaging and migraine: new connections?. Curr Opin Neurol. (2015) 28:265–70. 10.1097/WCO.0000000000000194 25887764 PMC4414904

[13] Bai L Tian J Zhong C Xue T You Y Liu Z. Acupuncture modulates temporal neural responses in wide brain networks: evidence from fMRI study. Mol Pain. (2010) 6:73. 10.1186/1744-8069-6-73 21044291 PMC2989943

[14] Huang W Pach D Napadow V Park K Long X Neumann J. Characterizing acupuncture stimuli using brain imaging with FMRI-a systematic review and meta-analysis of the literature. PLoS ONE. (2012) 7:e32960. 10.1371/journal.pone.0032960 22496739 PMC3322129

[15] Zang Y Jiang T Lu Y He Y Tian L. Regional homogeneity approach to fMRI data analysis. Neuroimage. (2004) 22:394–400. 10.1016/j.neuroimage.2003.12.030 15110032

[16] Turner BO Paul EJ Miller MB Barbey AK. Small sample sizes reduce the replicability of task-based fMRI studies. Commun Biol. (2018) 1:62. 10.1038/s42003-018-0073-z 30271944 PMC6123695

[17] Olesen J Dodick DW Ducros A. Headache Classification Committee of the International Headache Society (IHS). The international classification of headache disorders, 3rd edition (ICHD-3). Cephalalgia. (2018) 38:1–211. 10.1177/0333102417738202 29368949

[18] Chen J Zhao L Zheng H Li Y Yang M Chang X. Evaluating the prophylaxis and long-term effectiveness of acupuncture for migraine without aura: study protocol for a randomized controlled trial. Trials. (2013) 14:361. 10.1186/1745-6215-14-361 24171782 PMC3816544

[19] Kong J Gollub R Huang T Polich G Napadow V Hui K. Acupuncture de qi, from qualitative history to quantitative measurement. J Altern Complement ed. (2007) 13:1059–70. 10.1089/acm.2007.0524 18166116

[20] Ulett GA Han S Han JS. Electroacupuncture: mechanisms and clinical application. Biol Psychiatry. (1998) 44:129–38. 10.1016/S0006-3223(97)00394-6 9646895

[21] Diener HC Kronfeld K Boewing G Lungenhausen M Maier C Molsberger A. Efficacy of acupuncture for the prophylaxis of migraine: a multicentre randomised controlled clinical trial. Lancet Neurol. (2006) 5:310–6. 10.1016/S1474-4422(06)70382-9 16545747

[22] Facco E Liguori A Petti F Zanette G Coluzzi F De Nardin M. Traditional acupuncture in migraine: a controlled, randomized study. Headache. (2010) 48:398–407. 10.1111/j.1526-4610.2007.00916.x 17868354

[23] Linde K Allais G Brinkhaus B Manheimer E Vickers A White AR. Acupuncture for migraine prophylaxis. Cochrane Database Syst Rev. (2009) 1:CD001218. 10.1002/14651858.CD001218.pub2 PMC3099267 19160193

[24] Thach WT Goodkin HP Keating JG. The cerebellum and the adaptive coordination of movement. Annu Rev Neurosci. (1992) 15:403–42. 10.1146/annurev.ne.15.030192.002155 1575449

[25] Bastian AJ. Learning to predict the future: the cerebellum adapts feedforward movement control. Curr Opin Neurobiol. (2006) 16:645–9. 10.1016/j.conb.2006.08.016 17071073

[26] Schmahmann JD. Disorders of the cerebellum: ataxia, dysmetria of thought, and the cerebellar cognitive affective syndrome. J Neuropsychiatry Clin Neurosci. (2004) 16:367–78. 10.1176/jnp.16.3.367 15377747

[27] Timmann D Daum I. Cerebellar contributions to cognitive functions: a progress report after two decades of research. Cerebellum. (2007) 6:159–62. 10.1080/14734220701496448 17786810

[28] Ito M. Control of mental activities by internal models in the cerebellum. Nat Rev Neurosci. (2008) 9:304–13. 10.1038/nrn2332 18319727

[29] Helmchen C Mohr C Erdmann C Petersen D Nitschke MF. Differential cerebellar activation related to perceived pain intensity during noxious thermal stimulation in humans: a functional magnetic resonance imaging study. Neurosci Lett. (2003) 335:202–6. 10.1016/S0304-3940(02)01164-3 12531467

[30] Moulton EA Schmahmann JD Becerra L Borsook D. The cerebellum and pain: passive integrator or active participator? Brain Res Rev. (2010) 65:14–27. 10.1016/j.brainresrev.2010.05.005 20553761 PMC2943015

[31] Kros L Angueyra Aristizábal CA Khodakhah K. Cerebellar involvement in migraine. Cephalalgia. (2018) 38:1782–91. 10.1177/0333102417752120 29357683

[32] Mehnert J May A. Functional and structural alterations in the migraine cerebellum. J Cereb Blood Flow Metab. (2019) 39:730–9. 10.1177/0271678X17722109 28737061 PMC6446424

[33] Wang J-J Chen X Sah SK Zeng C Li Y-M Li N. Amplitude of low-frequency fluctuation (ALFF) and fractional ALFF in migraine patients: a resting-state functional MRI study. Clin Radiol. (2016) 71:558–64. 10.1016/j.crad.2016.03.004 27055741

[34] Amin FM Hougaard A Magon S Asghar MS Ahmad NN Rostrup E. Change in brain network connectivity during PACAP38-induced migraine attacks: a resting-state functional MRI study. Neurology. (2016) 86:1–8. 10.1212/WNL.0000000000002261 26674334

[35] Russo A Tessitore A Silvestro M Nardo FD Trojsi F Santo TD. Advanced visual network and cerebellar hyperresponsiveness to trigeminal nociception in migraine with aura. J Headache Pain. (2019) 20:46. 10.1186/s10194-019-1002-3 31053057 PMC6734311

[36] Seghier ML. The angular gyrus: multiple functions and multiple subdivisions. Neuroscientist. (2013) 19:43–61. 10.1177/1073858412440596 22547530 PMC4107834

[37] Yu ZB Peng J Lv YB Zhao M Xie B Liang ML. Different mean thickness implicates involvement of the cortex in migraine. Medicine (Baltimore). (2016) 95:e4824. 10.1097/MD.0000000000004824 27631235 PMC5402578

[38] Buckner RL Andrews-Hanna JR Schacter DL. The brain's default network. Ann NY Acad Sci. (2008) 1124:1–38. 10.1196/annals.1440.011 18400922

[39] Wang S Wang H Zhao D Liu X Yan W Wang M. Grey matter changes in patients with vestibular migraine. Clin Radiol. (2019) 74:898.e1–898.e5. 10.1016/j.crad.2019.07.015 31451181

[40] Ke J Yu Y Zhang X Su Y Wang X Hu S. Functional alterations in the posterior insula and cerebellum in migraine without aura: a resting-state MRI study. Front Behav Neurosci. (2020) 14:1–8. 10.3389/fnbeh.2020.567588 33132860 PMC7573354

[41] Zhang J Su J Wang M Zhao Y Zhang QT Yao Q. The sensorimotor network dysfunction in migraineurs without aura: a resting-state fMRI study. J Neurol. (2017) 264:1–10. 10.1007/s00415-017-8404-4 28154971

[42] de Kwaasteniet BP Rive MM Ruhé HG Schene AH Veltman DJ Fellinger L. Decreased resting-state connectivity between neurocognitive networks in treatment resistant depression. Front Psychiatry. (2015) 6:28. 10.3389/fpsyt.2015.00028 25784881 PMC4345766

[43] Lai CH Wu YT Hou YM. Functional network-based statistics in depression: theory of mind subnetwork and importance of parietal region. J Affect Disord. (2017) 217:132–7. 10.1016/j.jad.2017.03.073 28407556

[44] Chen C Yan M Yu Y Ke J Xu C Guo X. Alterations in regional homogeneity assessed by fMRI in patients with migraine without aura. J Med Syst. (2019) 43:298. 10.1007/s10916-019-1425-z 31352647

[45] Otti A Noll-Hussong M. Acupuncture-induced pain relief and the human brain's default mode network—an extended view of central effects of acupuncture analgesia. Forsch Komplementmed. (2012) 19:197–201. 10.1159/000341928 22964986

[46] Li K Zhang Y Ning Y Zhang H Liu H Fu C. The effects of acupuncture treatment on the right frontoparietal network in migraine without aura patients. J. Headache Pain. (2015) 16:33. 10.1186/s10194-015-0518-4 25916336 PMC4411327

[47] Zyloney CE Jensen K Polich G Loiotile RE Cheetham A LaViolette PS. Imaging the functional connectivity of the Periaqueductal Gray during genuine and sham electroacupuncture treatment. Mol Pain. (2010) 6:80. 10.1186/1744-8069-6-80 21080967 PMC2993660

